# Optimization design of solution preparation methods of three poorly soluble drugs of clinical use in infusions on orthogonal test and evaluation of stability study

**DOI:** 10.1016/j.bbrep.2025.102412

**Published:** 2025-12-15

**Authors:** Dan Jiang, Dongfei Fang, Zicen Wang, Huanqi Cun, Baoxia Fang, Fuchao Chen

**Affiliations:** a Sinopharm Dongfeng General Hospital, Hubei University of Medicine, Shiyan, Hubei, China; b School of Pharmaceutical Sciences, Hubei University of Medicine, Shiyan, Hubei, China

**Keywords:** Orthogonal test, Poorly soluble drugs, Particulate matter, Solution stability, Infusion preparation, Antibacterial drugs

## Abstract

**Purpose:**

To optimize the preparation methods of three intravenous poorly soluble antibacterial drugs (oxacillin sodium, cefmetazole sodium, and ciprofloxacin hydrochloride) and investigate their compatibility and stability in infusion.

**Methods:**

Taking the variety of solvents, volume of initial solvent, and oscillation time as influencing factors, and with the count of particulate matter and content uniformity in the preparation solution as the evaluation indices, the L_9_(3^4^) orthogonal test was used to optimize the preparation methods of three intravenous insoluble antibacterial drugs. Simulate clinical drug concentrations, mix each drug separately with 0.9 % sodium chloride (0.9 % NS) and 5 % glucose (5 % GS), and take samples at different time points under room temperature and refrigerator conditions to determine their appearance, pH value, and relative percentage of drug content.

**Results:**

The optimal preparation method for oxacillin sodium was as follows: it was pre-dissolved in 8 mL of 0.9 % NS and shaken for 90 s before dilution and preparation. The optimal preparation method for cefmetazole sodium was as follows: it was pre-dissolved in 8 mL of 5 % GS, and shaken for 120 s before dilution and preparation. The optimal preparation method for ciprofloxacin hydrochloride was as follows: it was pre-dissolved in 18 mL of 5 % GS, and oscillation for 120 s before dilution and preparation. The three drugs were combined with 0.9 % NS and 5 % GS, respectively, and remained relatively stable within 24 h.

**Conclusion:**

Optimal selection of the variety of solvents, the volume of the initial solvent, and the oscillation time in preparing poorly soluble antibiotics can improve the quality of the intravenous infusion. Stability experiments confirmed that the three drugs can remain stable for 24 h, ensuring safety during clinical intravenous infusion.

## Highlights

• Inadequate dissolution of insoluble injections may cause particles and dosing inaccuracy.
• Orthogonal test optimized IV formulation for three insoluble antimicrobial drugs.
• Compound stability study clarifies storage conditions for formulated drugs.

## Graphical abstract

**Figure:**
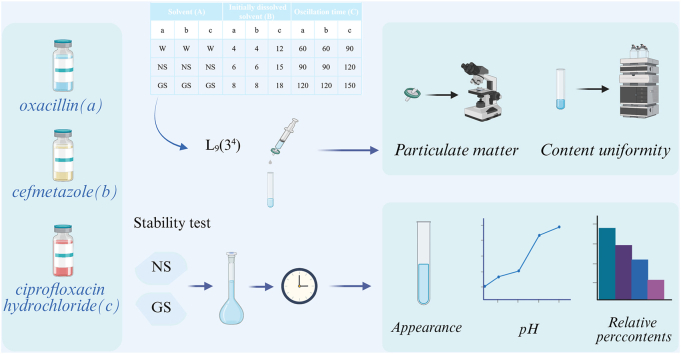

## 1 Introduction

Intravenous infusion serves as a critical route of administration in modern pharmacotherapy, playing an irreplaceable role in treatment of specific diseases and patient survival [1]. A survey demonstrated that intravenous fluid therapy administration is the most common invasive procedure in hospital settings [2]. Nevertheless, this administration method carries significant risks, including severe adverse reactions, that can endanger patient safety [3]. According to statistics, approximately 40 % of market drugs contain poorly soluble active ingredients [4]. Rodriguez Aller et al. [5] reported that 33 % of drugs included in the United States Pharmacopoeia(USP) and 75 % of compounds under development exhibit poor solubility. When mixing and dispensing these drugs, challenges such as difficulty in fully dissolving in a short time, complex operation, long time-consuming, or easy to produce foam often occur. These issues may increase contamination risks and the number of particulate matter in the infusion, potentially resulting in insufficient drug dosage [6].

Particles control in infusion preparation is a critical quality attribute of infusion products. Major pharmacopeias, including the Chinese Pharmacopoeia (ChP), USP, British Pharmacopoeia (BP), and Japanese Pharmacopoeia (JP), have established strict limits for subvisible particles (1–50 μm range) [7,8]. Once particles exceed the standard, they introduce non-metabolic foreign impurities into the body, which can lead to a series of clinical consequences, such as increased infusion-related reactions. Particulate matter contaminated infusions may have adverse effects in critically ill patients and are suspected to be responsible for unspecific inflammation that might lead to different organ manifestations [9]. Secondly, with prolonged storage time, the drug content may decrease due to chemical degradation following preparation, resulting in diminished therapeutic efficacy [10]. Furthermore, such degradation may lead to the formation of new compounds that possess toxic or adverse effects [11]. Consequently, the stability and allowable storage duration of the drug after dissolution are critical factors directly influencing the accuracy and safety of clinical administration.

Oxacillin sodium for injection is a semisynthetic penicillin derivative, administered as the sodium salt via parenteral route to treat the infections caused by methicillin-sensitive *Staphylococcus aureus* [12,13]. Cefmetazole, a second-generation cephalosporin, is effective against infections such as sepsis and acute bronchitis caused by *Staphylococcus aureus*. It also exhibits good antibacterial activity against certain cephalosporin-resistant strains and strong antibacterial activity against Staphylococcus, *Escherichia coli*, and Klebsiella [14,15]. Ciprofloxacin is a broad-spectrum antibiotic indicated for the treatment of pulmonary diseases including chronic obstructive pulmonary disease and cystic fibrosis, as well as infections caused by susceptible bacteria in the urinary, reproductive, respiratory, and gastrointestinal systems [16]. During the preparation of poorly soluble antimicrobial drugs, pharmacists frequently encountered challenges with three drugs their common use and complex reconstitution characteristics. The initial dissolution time was long, there were ambiguous preparation methods in the package insert, no unified judgment standard for complete dissolution, and no unified and standard operation procedure for the preparation. Therefore, an L9(3) [4] orthogonal test was employed to evaluate the effects of various solvents, initial solvent volume, and oscillation time on the dissolution of these three antibacterial drugs in our study. The quality of infusion preparation was evaluated based on particulate matter counts and content uniformity. In addition, this work aims to establish a foundation for further research on quality control in antimicrobial infusion preparation. Additionally, stability experiments were conducted by simulating clinical concentrations to investigate the stability of the three drugs during the infusion process, thereby ensuring their safety in clinical intravenous administration.

## 2 Materials and methods

### 2.1 Materials and reagents

Oxacillin standard (batch number: 130482–202303, purity 89.8 %), Cefmetazole standard (batch number: 130580–202002, purity 99.4 %), and Ciprofloxacin (batch number: 130451–201904, purity 83.1 %) were obtained from National Institutes for Food and Drug Control (Beijing, China). Oxacillin Sodium for Injection (batch number: 230906B, 1.0 g) (Sichuan pharmaceutical preparation Co., Ltd.), Cefmetazole Sodium for Injection (batch number: G230618, 1.0 g) (Zhejiang Yatai Pharmaceutical Co., Ltd.), Ciprofloxacin Hydrochloride for Injection (batch number: 2312015, 0.4 g) (Hainan Herui Pharmaceutical Co., Ltd.). All of the above medicines were purchased from Sinopharm Holding Company Limited (Hubei, China). HPLC-grade acetonitrile and methanol were purchased from Tianjin Kermel Chemical Reagent Co., Ltd. (Tianjin, China). The sterilized water for injection was purchased from Hubei Kelun Pharmaceutical Co., Ltd., 0.9 % sodium chloride (0.9 % NS) injection and 5 % glucose (5 % GS) injection were purchased from Otsuka Pharmaceutical Co., Ltd (Dalian, China).

### 2.2 Instrumentation

The analysis was conducted using an Agilent 1260, High-performance Liquid Chromatography (HPLC) (Agilent, America) equipped with a variable wavelength detector. The chromatographic column was Agilent ZORBAX SB-C_18_, 150 × 4.6 mm with 5 μm particle size, supplied by Agilent. The chromatographic data were processed using Open Lab CDS 2. The pH was measured with a pH meter (PHS–3C, INESA China). MS105DU electronic balance (Mettler Toledo, Switzerland). The particulate matter was observed with a biological microscope (CX41 RF, Olympus) equipped with a UIS2 optical system. The type of low constant incubator was SPX-50B. The Osmometer was measured by the Model 3250 Osmometer.

### 2.3 Chromatographic conditions

According to the method specified in the Pharmacopoeia of the People's Republic of China, the detection of Oxacillin was carried out using acetonitrile and 0.04 mol L^−1^ potassium dihydrogen phosphate (phosphoric acid to adjust pH to 5.0) (25: 75) as mobile phase with the flow rate of 0.8 mL min^−1^ for 15 min. The detection of cefmetazole was carried out using ammonium dihydrogen phosphate, tetrahydrofuran, and methanol (phosphoric acid to adjust pH to 4.5) (730: 12.5: 300) as mobile phase with the flow rate of 1.0 mL min^−1^ for 15 min. Ciprofloxacin was detected using acetonitrile and 0.025 mol L^−1^ phosphoric acid (triethylamine to adjust pH to 3.0) (13: 87) as mobile phase with the flow rate of 1.5 mL min^−1^ for 15 min. The detection of the wavelengths of oxacillin, cefmetazole, and ciprofloxacin were 225 nm, 254 nm, and 278 nm, respectively. All injection volumes were fixed at 20 μL, and separations were performed at 30 °C.

### 2.4 Orthogonal test design

Following the instructions of the three poorly soluble antimicrobials, the respective formulation method was determined. According to previous work experience, the variety of solvent (factor A), volume of initially dissolved solvent (factor B), and oscillation time (factor C) were the main factors affecting the effect of drug blending. The count of particulate matter (CPM) and content uniformity (CU) in the drug solution after solution were taken as the index, and the blending efficiency was taken into account according to the dissolution characteristics and volume of each drug bottle. The level values of each factor in the orthogonal test for each drug were determined. Table 1 shows the orthogonal test L_9_(3^4^) parameters. Factor D was blank, and no measures were taken, which was only used for statistical analysis. Each treatment was performed in triplicate.

**Table 1:** Factors and levels of orthogonal test.

Levels	Factor A			Factor B (mL)			Factor C (s)		
	a	b	c	a	b	c	a	b	c
1	W	W	W	4	4	12	60	60	90
2	NS	NS	NS	6	6	15	90	90	120
3	GS	GS	GS	8	8	18	120	120	150

Note: A. Variety of solvent; B. volume of initially dissolved solvent; C. oscillation time; a. Oxacillin Sodium; b. Cefmetazole Sodium; c. Ciprofloxacin hydrochloride (for Table 1, Table 2, Table 3, Table 4); W. sterilized water for injection; NS. 0.9%Sodium Chloride Injection; GS. 5 % Glucose Injection.

#### 2.4.1 Particulate matter

The solution preparation was always carried out by professional pharmaceutical personnel according to aseptic procedures, and the intravenous drugs were mixed and prepared under Class 100 cleanroom conditions. The poorly soluble particle inspection method was used after mixing, according to General Rule 0903 of the 2020 edition of the Pharmacopoeia of the People's Republic of China (Part IV). The solution was extracted using an injection syringe and slowly injected along the inner wall into the filter, which contained a microporous membrane with a pore size of 25 mm. Stand it for 1 min, slowly filter it until the membrane is almost dry, then use 5 mL of water for washing until the filter membrane is nearly dry. The process of washing and filtering the solution should be continued until the filter membrane is nearly dry. The filter membrane was then transferred onto a flat dish using flat-headed tweezers. The lid was gently lifted to allow the filter membrane to dry properly, and the flat dish was closed. The dish was placed on the stage of the microscope, the incident light was adjusted, and the magnification was set to 100 times for microscopic measurement. The microscope was then adjusted until the filter membrane grid was visible, the coordinate axis was moved, and the number of particles with a diameter greater than 10 μm in the effective filtration area was measured. The average value of the measurement results from three test samples was then calculated. The CPM was counted using the microscopic counting method after the poorly soluble antibacterial drugs had been dissolved.

#### 2.4.2 Content uniformity test

The mixture was carried out according to the orthogonal experimental design table. When each experimental plan was completed, the solution of the upper part, the middle part and the lower part of the filling bottle were extracted 1 mL, respectively. According to the above chromatographic conditions, the three parts of the liquid after each experiment were diluted, and the content was tested.

The CU was calculated by the following formula (1).

(1) $$CU\left(RSD\%\right)=s/\bar{x}\times 100\%$$

Where s is the standard deviation of the concentration of upper, middle, and lower liquid, $\bar{x}$ is the average of the concentration of upper, middle, and lower liquid.

### 2.5 Compatibility stability investigation

Compatibility assessments of three poorly soluble antimicrobials (oxacillin sodium, cefmetazole sodium, and ciprofloxacin hydrochloride) were conducted according to clinical dosing regimens. Stability profiles in 0.9 % sodium chloride injection (NS) and 5 % glucose solution (GS) were evaluated under 25 ± 1 °C and 4 ± 1 °C. The stability of the three antibacterial drugs in different solvents was investigated by observing the appearance, pH, and content changes of the compatibility solution at predetermined intervals (0, 2, 4, 8, 12, 24 h).

#### 2.5.1 Preparation of compatibility solution

According to the clinical drug concentration, standards of oxacillin sodium, cefmetazole sodium, and ciprofloxacin hydrochloride were precisely weighed (the sample size was corrected according to the purity). The three drugs were prepared into three kinds of compatibility test solutions with different concentrations by diluting with 0.9 % NS and 5 % GS. The concentrations of compatibility solutions were oxacillin concentrations of 16.0 mg mL^−1^, 8.0 mg mL^−1^, and 4.0 mg mL^−1^, cefmetazole concentrations of 16.0 mg mL^−1^, 8.0 mg mL^−1^, and 4.0 mg mL^−1^, and ciprofloxacin concentrations of 0.5 mg mL^−1^, 1.5 mg mL^−1^, and 2.5 mg mL^−1^.

#### 2.5.2 Preparation of test solution

(1) Oxacillin sodium: 250 μL of oxacillin sodium was placed in a 25 mL volumetric flask and diluted to 25 mL with the mobile phase. Subsequently, 20 μL was drawn for the determination.
(2) Cefmetazole sodium: 625 μL of cefmetazole sodium was placed in a 25 mL volumetric flask and diluted to 25 mL with the mobile phase. Subsequently, 20 μL was drawn for the determination.
(3) Ciprofloxacin hydrochloride: 500 μL of ciprofloxacin hydrochloride was placed in a 25 mL volumetric flask and diluted to 25 mL with the mobile phase. Subsequently, 20 μL was drawn for the determination.

#### 2.5.3 Stability of the compatibility solution

Three drugs of compatibility solution were stored at 25 ± 1 °C, with lighting and refrigerator (2–8 °C). Recorded changes in the appearance, pH, osmotic pressure, and content of the compatibility solution at predetermined intervals (0, 2, 4, 8, 12, 24 h). Diluted the different compatible liquids according to the conditions in 2.5.2 and tested the content. The relative percentage content of each drug was calculated with the content of the compatibility solution at 0 h as 100 %.

### 2.6 Analysis of data

According to the requirements in the Pharmacopoeia of the People's Republic of China, the counting of particulate matter is the primary indicator. The overall score was calculated by formula (2), whereas 0.5 is the weight coefficient of CU and 0.5 is the weight coefficient of CPM.

(2) Overallscore=CPM×0.5+CU×0.5

The experiments were performed three times unless otherwise mentioned. GraphPad Prism 9.0 was used to draw the figures. SPSS 26.0 software was applied, and measurement data were presented as $\bar{x}\pm s$. Analysis of variance was used, and *P* < 0.05 was considered statistically significant.

## 3 Results

### 3.1 Methodological validation of HPLC

**Fig. 1:**
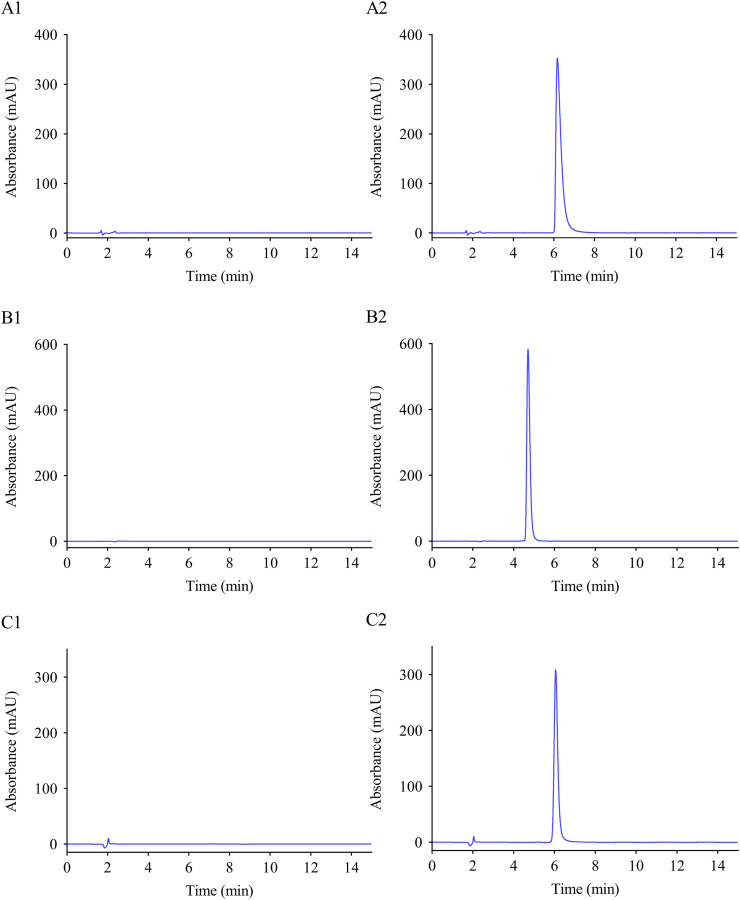
Chromatograms of the three drugs Note: A. Oxacillin Sodium; B. Cefmetazole Sodium; C. Ciprofloxacin hydrochloride; 1. Blank control; 2. Standard solution.

The results of HPLC for the linear range and linear relationship, precision, stability, and recovery rate of the three antibacterial drugs are shown in Table 2. The chromatograms of these drugs are shown in Fig. 1. The impurity peak did not interfere with the determination of the main component; the degree of separation was greater than 1.5. The retention times of oxacillin sodium, cefmetazole sodium, and ciprofloxacin hydrochloride were 6.3, 4.7, and 5.6 min, respectively. As shown in the table, the relative standard deviation (RSD) of the precisions and drug concentrations at different time points for the three drugs was below 2.0 %, indicating good precision of the instrument. The prepared sample solution remained stable for 8 h at 25 ± 1 °C lighting. The recoveries of analytes ranged from 99.6 % to 100.6 %, and the RSD was less than 2.0 %, meaning that the recovery rate of the method was good.

**Table 2:** Methodological validation parameters of the HPLC method.

Drug	Linear equation	r	Linear Range (μg∙mL^−1^)	Stability RSD (%)	Repeatability RSD (%)	Recovery		Precision	
						X (%)	RSD (%)	Intra-Day (%)	Inter-Day (%)
a	y = 62.917x+325.22	0.9993	25–200	0.58	0.52	100.60	0.90	0.22	1.10
b	y = 27.572x+967.01	0.9999	50–400	0.64	0.91	98.42	1.71	0.62	0.91
c	y = 10.1647x-12.043	0.9996	5–80	1.19	1.31	99.61	1.20	0.78	1.27

Note: a. Oxacillin Sodium; b. Cefmetazole Sodium; c. Ciprofloxacin hydrochloride.

### 3.2 Optimization of the infusion formulation

The orthogonal test, as a scientific and systematic method, was employed to optimize the preparation conditions for the three antimicrobial powder injections [17]. In this study, the count of particulate matter and content uniformity were selected as observation indices, and a comprehensive scoring was used to assess the quality of infusion preparation.

**Fig. 2:**
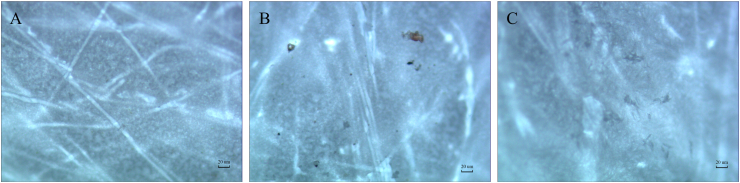
**Microscopic observation results ( × 100)** Note: A. Blank (none drugs) B. When the solution is incompletely dissolved C. When the solution is completely dissolved.

The observation results under the microscope (100 times magnification) of the blank group (without drugs) are shown in Fig. 2A. The observation results when the drug was incompletely dissolved were shown in Fig. 2B, and the observation results when the drug was completely dissolved were shown in Fig. 2C. Orthogonal test results, K and R values are displayed in Table 3, and analysis of variance is shown in Table 4. In the Orthogonal test, the K value represents the sum of the test results corresponding to the horizontal number on any column. The R value was the range, and it was calculated as max{K1, K2, K3} - min{K1, K2, K3}.

**Table 3:** Results of L_9_(3^4^) orthogonal test.

Number		Factors				a			b			c		
		A	B	C	D	CPM ($\bar{x}$*±s*)	CU (%)	score	CPM ($\bar{x}$*±s*)	CU (%)	score	CPM ($\bar{x}$*±s*)	CU (%)	score
1		1	1	1	1	66.00 ± 4.16	2.19 ± 0.33	34.10	17.75 ± 0.90	1.93 ± 0.09	9.84	3.58 ± 0.38	1.19 ± 0.14	2.39
2		1	2	2	2	16.80 ± 1.59	1.28 ± 0.33	9.04	15.33 ± 0.33	1.03 ± 0.11	8.18	1.87 ± 0.13	2.08 ± 0.15	1.97
3		1	3	3	3	10.25 ± 0.82	1.42 ± 0.13	5.84	6.88 ± 0.88	1.12 ± 0.12	4.00	0.67 ± 0.12	1.92 ± 0.08	1.29
4		2	1	2	3	37.83 ± 1.13	1.19 ± 0.16	19.51	17.25 ± 0.66	3.38 ± 0.21	10.32	10.8 ± 1.37	8.43 ± 0.59	9.63
5		2	2	3	1	16.27 ± 1.51	1.08 ± 0.14	8.67	12.33 ± 0.76	2.80 ± 0.14	7.57	12.9 ± 1.90	3.53 ± 0.39	8.23
6		2	3	1	2	8.17 ± 0.80	0.85 ± 0.15	4.51	3.04 ± 0.44	0.98 ± 0.04	2.01	11.5 ± 1.71	4.19 ± 0.57	7.84
7		3	1	3	2	47.08 ± 3.56	1.13 ± 0.10	24.11	11.17 ± 0.88	2.16 ± 0.24	6.67	3.00 ± 0.24	0.26 ± 0.06	1.63
8		3	2	1	3	17.27 ± 1.40	1.33 ± 0.26	9.30	4.89 ± 0.79	0.45 ± 0.08	2.67	1.20 ± 0.11	1.02 ± 0.90	1.11
9		3	3	2	1	7.71 ± 0.85	1.09 ± 0.12	4.40	5.42 ± 0.51	0.47 ± 0.12	2.95	0.87 ± 0.16	1.85 ± 0.14	1.34
a	K_1_	16.327	25.907	15.970	15.723	B > A > C A2 B3 C2								
	K_2_	10.897	9.003	10.983	12.553									
	K_3_	12.603	4.917	12.873	11.550									
	R	5.430	20.990	4.987	4.173									
b	K_1_	6.673	8.943	4.173	6.120	B > C > A A3 B3 C1								
	K_2_	7.300	6.140	7.817	5.620									
	K_3_	4.097	2.987	6.080	6.330									
	R	3.203	5.956	3.644	0.710									
c	K_1_	1.883	4.55	3.78	3.987	A > B > C A3 B3 C3								
	K_2_	8.567	3.770	4.313	3.813									
	K_3_	1.360	3.490	3.717	4.010									
	R	7.207	1.060	0.596	0.197									

Note: A. Variety of solvent; B. volume of initially dissolved solvent; C. oscillation time; a. Oxacillin Sodium; b. Cefmetazole Sodium; c. Ciprofloxacin hydrochloride.

**Table 4:** Analysis of variance.

Drug	Factor	Sum of squares	Freedom	Mean square error	F	*p*
a	A	46.192	2	23.096	1.622	0.381
	B	742.946	2	371.473	26.082	0.037
	C	37.984	2	18.992	1.333	0.429
b	A	17.485	2	8.742	6.667	0.13
	B	53.256	2	26.628	20.306	0.047
	C	7.983	2	3.991	3.044	0.247
c	A	96.893	2	48.446	1407.349	0.001
	B	1.797	2	0.898	26.096	0.037
	C	0.648	2	0.324	9.411	0.096

Note: A. Variety of solvent; B. volume of initially dissolved solvent; C. oscillation time; a. Oxacillin Sodium; b. Cefmetazole Sodium; c. Ciprofloxacin hydrochloride.

From Table 3, Table 4, ANOVA results demonstrated that the oscillation time had no significant effect on the total scores of the three drugs (*P* > 0.05), whereas the initially dissolution solvent volume significantly affected the total score of the three drugs (*P* < 0.05), with solvent varieties showing a significant effect solely for ciprofloxacin hydrochloride (*P* < 0.05). Combined with the intuitive analysis results, the factors affected the three drugs in the following order of significance: B > A > C for oxacillin sodium, B > C > A for cefmetazole sodium, and A > B > C for ciprofloxacin hydrochloride. Accordingly, the optimal preparation formulations were determined as follows: for oxacillin sodium, 8 mL solvent (sterilized water for injection, 0.9 % sodium chloride injection, or 5 % glucose injection) was pre-dissolved, and the mixture was completed after oscillation and dilution. For cefmetazole sodium, pre-dissolution in 8 mL of the same solvent options as oxacillin sodium, followed by oscillation and dilution. And for ciprofloxacin hydrochloride, pre-dissolution in 18 mL of 5 % glucose injection, followed by oscillation and dilution.

### 3.3 Results of compatibility stability investigation

**Fig. 3:**
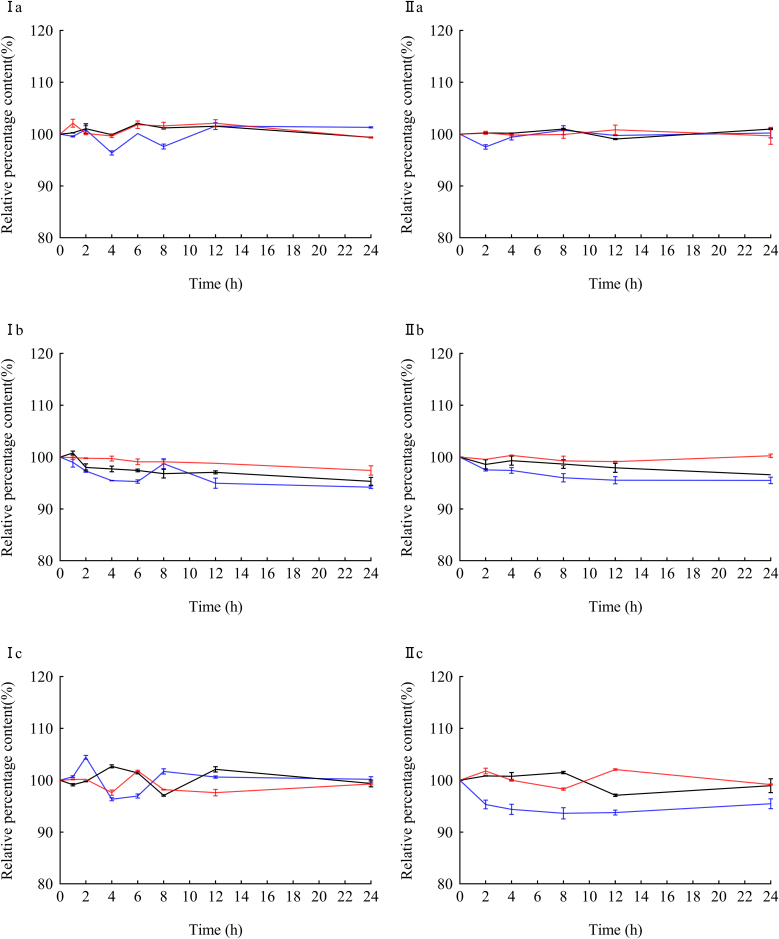
The relative percentage content of different concentrations of the three drugs varied in 0.9 % sodium chloride Injection. Note: a. Oxacillin Sodium; b. Cefmetazole Sodium; c. Ciprofloxacin hydrochloride (for Fig. 3, Fig. 4); Ⅰ. 25 ± 1 °C, light; Ⅱ. refrigerator (2–8 °C), protect from light (for Fig. 2, Fig. 3); **−−−**. high concentration; **−−−**. mid concentration; **−−−**. low concentration.

**Fig. 4:**
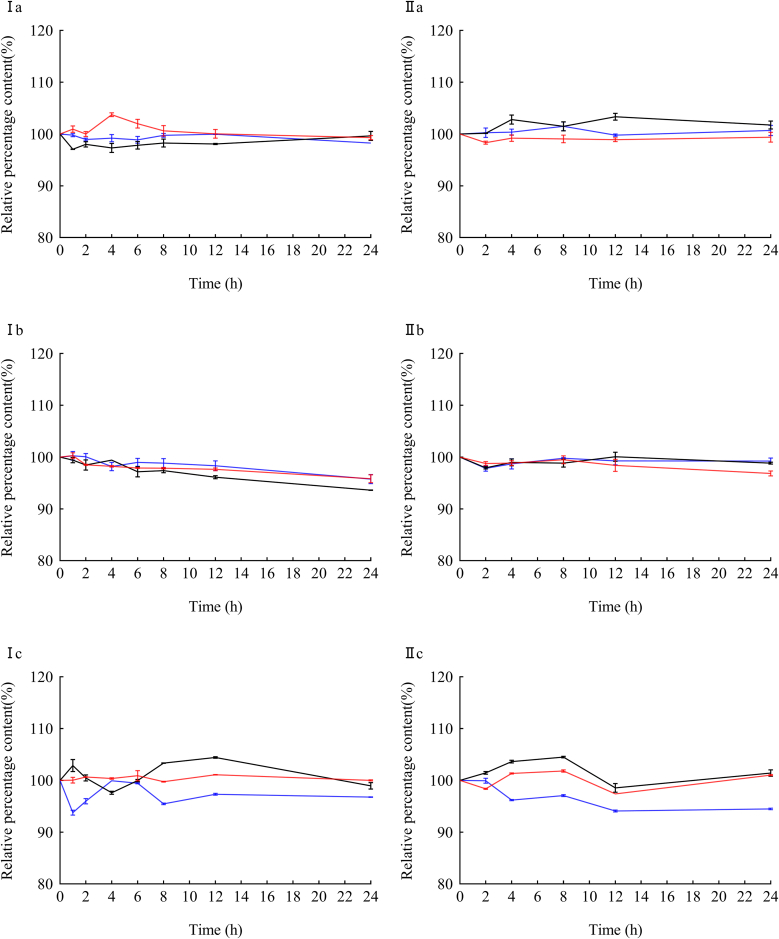
The relative percentage content of different concentrations of the three drugs varied in 5 % Glucose Injection.

**Fig. 5:**
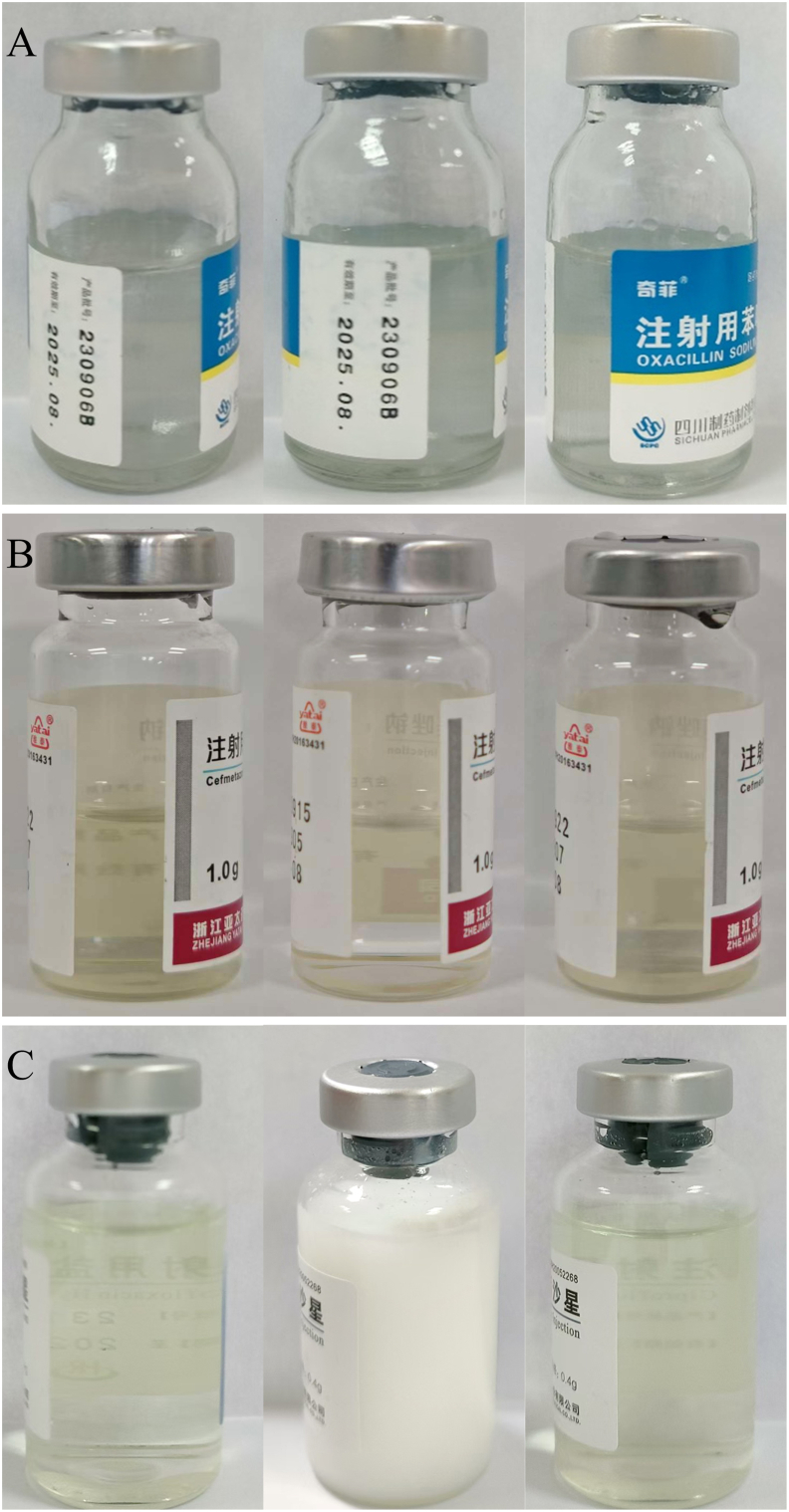
Three drugs are dissolved in different solvents Note: A. Oxacillin Sodium was dissolved separately in injection-sterilized water, 0.9 % sodium chloride injection, and 5 % glucose injection (from left to right), with each volume being 8 mL.B. Cefmetazole Sodium was dissolved separately in injection-sterilized water, 0.9 % sodium chloride injection, and 5 % glucose injection (from left to right), with each volume being 6 mL C. Ciprofloxacin hydrochloride was dissolved separately in injection-sterilized water, 0.9 % sodium chloride injection, and 5 % glucose injection (from left to right), with each volume being 18 mL.

The stability test results showed that the mixture of the three drugs with 0.9 % NS and 5 % GS was colorless and transparent, without precipitation and bubble formation. The pH values of oxacillin sodium, cefmetazole Sodium, and ciprofloxacin hydrochloride were between 5.5 and 6.1, 4.0 and 4.8, and 4.0 and 4.6, with no obvious fluctuation. Under the corresponding detection conditions, the relative percentage contents of various concentrations of drugs were close to 100 % and remained relatively stable, as shown in Fig. 3, Fig. 4. The dissolution conditions of the three drugs in different solvents were shown in Fig. 5. The relative standard deviation (RSD) values of the osmotic pressure for different concentrations of the drug are less than 2 %, as shown in Table 5, Table 6. Indicates that all three drugs are stable for 24 h at room temperature and refrigerated conditions.

**Table 5:** The osmotic pressure changes of different concentrations of the three drugs varied in 0.9 % sodium chloride injection over 24 h (‾*x* ± *s*,n = 3).

Drug	Concentrations	Concentrations	Time (h)						
			0	2	4	6	8	12	24
a	High	Ⅰ	360 ± 0.58	361 ± 0.58	360 ± 1.15	360 ± 1.15	359 ± 0.58	360 ± 0.58	361 ± 0.58
		Ⅱ	360 ± 0.58	361 ± 0.58	360 ± 1.00	360 ± 0.58	360 ± 0.58	361 ± 0.58	359 ± 0.58
	Mid	Ⅰ	323 ± 0.58	322 ± 0.58	322 ± 0.58	321 ± 0.58	321 ± 1.00	321 ± 1.15	320 ± 0.58
		Ⅱ	323 ± 0.58	322 ± 0.58	322 ± 0.58	321 ± 0.58	321 ± 1.00	321 ± 1.15	320 ± 0.58
	Low	Ⅰ	304 ± 0.58	304 ± 1.00	302 ± 1.15	303 ± 1.53	301 ± 1.00	301 ± 1.00	301 ± 0.58
		Ⅱ	304 ± 0.58	305 ± 0.58	305 ± 1.15	304 ± 0.58	303 ± 0.58	302 ± 0.58	301 ± 0.58
b	High	Ⅰ	340 ± 0.58	340 ± 0.58	337 ± 0.58	338 ± 0.58	337 ± 0.58	337 ± 0.58	336 ± 1.53
		Ⅱ	341 ± 0.58	341 ± 0.58	340 ± 0.58	340 ± 0.58	339 ± 0.58	338 ± 0.58	338 ± 1.53
	Mid	Ⅰ	316 ± 1.00	315 ± 0.58	315 ± 0.58	314 ± 1.00	313 ± 0.58	312 ± 0.58	311 ± 0.58
		Ⅱ	316 ± 1.00	316 ± 0.58	315 ± 0.58	314 ± 0.58	312 ± 1.00	312 ± 0.58	312 ± 0.58
	Low	Ⅰ	300 ± 0.58	300 ± 0.58	299 ± 0.58	296 ± 0.58	295 ± 0.58	295 ± 1.15	294 ± 0.58
		Ⅱ	300 ± 0.58	301 ± 0.58	300 ± 0.58	298 ± 1.00	297 ± 1.00	297 ± 1.52	296 ± 0.58
c	High	Ⅰ	307 ± 0.58	306 ± 0.58	307 ± 1.00	307 ± 0.58	306 ± 0.58	305 ± 0.58	305 ± 1.15
		Ⅱ	307 ± 0.58	306 ± 0.58	306 ± 0.58	306 ± 0.58	307 ± 0.58	306 ± 0.58	307 ± 0.58
	Mid	Ⅰ	284 ± 0.58	285 ± 0.58	284 ± 0.58	284 ± 1.00	283 ± 1.00	283 ± 1.00	282 ± 1.00
		Ⅱ	284 ± 0.58	286 ± 0.58	285 ± 0.58	284 ± 1.00	284 ± 0.58	283 ± 0.58	284 ± 0.58
	Low	Ⅰ	272 ± 0.58	273 ± 0.58	273 ± 0.58	273 ± 0.58	273 ± 1.00	272 ± 0.58	271 ± 1.00
		Ⅱ	272 ± 0.58	273 ± 0.58	272 ± 1.00	272 ± 0.58	273 ± 1.53	273 ± 1.00	273 ± 1.15

Note: a. Oxacillin Sodium; b. Cefmetazole Sodium; c. Ciprofloxacin hydrochloride; Ⅰ. 25 ± 1 °C, light; Ⅱ. refrigerator (2–8 °C), protect from light.

**Table 6:** The osmotic pressure changes of different concentrations of the three drugs varied in 5 % glucose injection over 24 h (‾*x* ± *s*,n = 3).

Drug	Concentrations	Concentrations	Time (h)						
			0	2	4	6	8	12	24
a	High	Ⅰ	341 ± 0.58	339 ± 0.58	338 ± 0.58	339 ± 0.58	338 ± 0.58	337 ± 1.53	334 ± 1.15
		Ⅱ	341 ± 0.58	340 ± 0.58	340 ± 0.58	339 ± 0.58	340 ± 0.58	339 ± 0.58	338 ± 1.00
	Mid	Ⅰ	309 ± 1.00	308 ± 0.58	306 ± 1.15	306 ± 1.00	304 ± 0.58	303 ± 0.58	303 ± 1.00
		Ⅱ	309 ± 1.00	308 ± 0.58	307 ± 0.58	306 ± 0.58	306 ± 0.58	305 ± 0.58	303 ± 0.58
	Low	Ⅰ	284 ± 0.58	284 ± 0.58	284 ± 0.58	283 ± 0.58	283 ± 0.58	281 ± 0.58	280 ± 0.58
		Ⅱ	284 ± 0.58	284 ± 0.58	283 ± 0.58	283 ± 0.58	281 ± 0.58	281 ± 1.00	280 ± 0.58
b	High	Ⅰ	323 ± 0.58	323 ± 0.58	323 ± 0.58	323 ± 1.00	324 ± 0.58	323 ± 0.58	322 ± 0.58
		Ⅱ	323 ± 0.58	322 ± 0.58	323 ± 0.58	323 ± 1.00	323 ± 0.58	322 ± 1.00	322 ± 0.58
	Mid	Ⅰ	291 ± 0.58	292 ± 0.58	292 ± 0.58	291 ± 0.58	293 ± 0.58	292 ± 1.00	291 ± 0.58
		Ⅱ	291 ± 0.58	291 ± 0.58	291 ± 0.58	292 ± 0.58	293 ± 1.00	293 ± 1.15	292 ± 0.58
	Low	Ⅰ	279 ± 0.58	278 ± 0.58	278 ± 1.00	277 ± 0.58	276 ± 1.53	275 ± 0.58	274 ± 0.58
		Ⅱ	279 ± 0.58	278 ± 0.58	279 ± 0.58	278 ± 1.00	278 ± 0.58	277 ± 0.58	276 ± 1.00
c	High	Ⅰ	298 ± 1.15	297 ± 0.58	298 ± 1.00	298 ± 0.58	297 ± 0.58	296 ± 0.58	296 ± 0.58
		Ⅱ	298 ± 1.15	298 ± 0.58	298 ± 0.58	298 ± 0.58	298 ± 1.15	297 ± 1.00	296 ± 0.58
	Mid	Ⅰ	278 ± 0.58	277 ± 0.58	276 ± 0.58	277 ± 0.58	277 ± 1.00	277 ± 0.58	276 ± 0.58
		Ⅱ	278 ± 0.58	280 ± 1.00	280 ± 0.58	279 ± 1.53	278 ± 0.58	279 ± 0.58	278 ± 0.58
	Low	Ⅰ	264 ± 0.58	266 ± 1.00	264 ± 0.58	263 ± 0.58	263 ± 0.58	261 ± 1.00	261 ± 0.58
		Ⅱ	264 ± 0.58	264 ± 1.00	264 ± 0.58	263 ± 0.58	263 ± 1.00	263 ± 0.58	264 ± 0.58

Note: a. Oxacillin Sodium; b. Cefmetazole Sodium; c. Ciprofloxacin hydrochloride; Ⅰ. 25 ± 1 °C, light; Ⅱ. refrigerator (2–8 °C), protect from light.

## 4 Discussion

Intravenous infusion is a widely used method of drug delivery in clinical practice; yet it entails the risk of particulate contamination. When the particle size of particulate matter exceeds a certain size, or the number exceeds a certain limit, it will accumulate in the human body. It will cause some harm to the human body, may cause pulmonary edema, phlebitis, heat reaction, thrombosis, tissue necrosis, and other diseases, and even cause allergic reactions in serious cases. Therefore, it is imperative to monitor and stringently control the level of particulate matter in the injection [[18], [19], [20]]. Optimizing the dispensing method of powder injection can effectively reduce the production of particulate matter, and at the same time, make the drug completely dissolve in the initial dissolution as much as possible to ensure the accuracy of the infusion dose. Several studies have proved that the orthogonal test is a scientific method that allows for the selection of the best experimental conditions for the combination of optimal factors [[21], [22], [23]]. The orthogonal test has several advantages, including reducing the number of testing treatments and facilitating the analysis of scientific results. The present study evaluated all the selected factors using an orthogonal L_9_(3^4^) test design. In the preliminary investigation, through monitoring the residual liquid in the bottle and the content of the finished infusion product, we found that the residual liquid of oxacillin, cefmetazole, and ciprofloxacin hydrochloride in the preparation process was more, and the drug content of the finished product was lower. Oxacillin, cefmetazole, and ciprofloxacin hydrochloride are considered to be poorly soluble powder injections in clinical settings, which causes great inconvenience to the dispensing personnel.

Powder injection for injection was prepared by dissolving it in a suitable injection solvent, and then it was injected into an intravenous infusion bottle to dilute it for clinical intravenous infusion until the drug was fully dissolved and clarified. If there is no special requirement for the solvent for injection, the appropriate intravenous infusion solution shall be selected for dissolution according to the doctor's advice. For example, the specification for oxacillin sodium for injection describes that when utilized for intramuscular injection, each 0.5 g is dissolved in 2.8 mL of sterilized water for injection, with no provision for use in intravenous infusion. However, in clinical practice, 5 mL of 0.9 % NS is frequently used as the initial solvent. In some cases, the instructions for a particular drug do not explicitly provide the amount of solvent to be used. Instead, they may stipulate the desired final concentration of the drug after completion of the preparation process and the duration of the infusion. For example, ciprofloxacin hydrochloride is described in the instructions for intravenous administration. Before its administration, the drug was dissolved in 200 mL of either 0.9 % NS or 5 % GS.

This study used three commonly used solvents in clinical practice as initial solvents for experiments, and the results showed that using 0.9 % NS or 5 % GS as initial solvents could better dissolve these three drugs. The stability study results showed that the three drugs remained relatively stable for 24 h at room temperature or refrigerated conditions in 0.9 % NS and 5 % GS. The quantity of solvent and the concentration of the drug are important parameters for controlling the quality of infusion. Secondly, the amount of solvent used will affect the quality of the finished infusion. This study further verified this statement through orthogonal experiments, and the results of variance analysis showed that the volume of solvent had a significant impact on all three drugs. In addition, the dissolution volume can also affect the measurement of equilibrium solubility, as reducing the volume of the solvent system during shaking can accelerate the saturation rate, allowing the solution to reach equilibrium faster. Therefore, when preparing insoluble intravenous drugs, it is advisable to avoid using excessive initial doses of solvent, as this can reduce both dissolution and saturation efficiency, and may also hinder the workflow efficiency of dispensing personnel [24]. A limitation of this study was that it did not evaluate the potential impact of packaging materials (e.g., glass bottles, flexible bags) on the compatibility stability of the infusion. Future studies should closely replicate clinical infusion scenarios to assess the stability of the prepared solutions in these different containers.

## 5 Conclusion

In this study, the particulate matter count and content uniformity in the concentrated solution in the mixed were determined to ensure stringent control of particulate levels in poorly soluble intravenous drugs. Through orthogonal testing, the optimum mixing conditions for the three drugs were determined as A2B3C2, A3B3C1, and A3B3C3, respectively. The established optimal preparation method can be promoted as a standard protocol. This standardization not only sets formulation criteria for poorly soluble intravenous drugs but also reduces particulate matter in the final infusion, thereby enhancing preparation efficiency. Simultaneously, as confirmed by stability testing, the three drugs can remain stable for 24 h in 0.9 % NS and 0.5 GS.

## Research ethics and consent

Not applicable.

## Funding

This study was supported by the Natural Science Foundation of Hubei Province, China (grant number 2024AFD097) and the Technology Key Program of Shiyan, China (grant number 24Y153).

## CRediT authorship contribution statement

**Dan Jiang:** Conceptualization, Validation, Writing – original draft. **Dongfei Fang:** Conceptualization, Validation, Writing – original draft. **Zicen Wang:** Data curation. **Huanqi Cun:** Data curation. **Baoxia Fang:** Methodology, Supervision, Writing – review & editing. **Fuchao Chen:** Methodology, Supervision, Writing – review & editing.

## Declaration of competing interest

The authors declare that they have no known competing financial interests or personal relationships that could have appeared to influence the work reported in this paper.

## Figures and Tables

**Table:** 

Abbreviations	Full name
0.9 % NS	0.9 % sodium chloride
5 % GS	5 % glucose
HPLC	High-Performance Liquid Chromatography
RSD	Relative Standard Deviation
CPM	count of particulate matter
CU	content uniformity

## Data Availability

All data generated or analyzed in this study are included in the published article. All other data are available from the corresponding author upon request.
